# Excision of the Facial Bones

**Published:** 1890-03

**Authors:** 


Dr. Pean presented before the Academie de Medecine a case of total excision of the facial bones for multiple osseous tumors of the bones of the face. The patient was a woman 32 years of age, in whom the sphenoid, the two maxillary and the malar bones were invaded by an osteo-fibroma, due to odontomia. The affection dated nine years. In 1884, a surgeon resected the right superior maxillary, but the disease soon recurred in the corresponding bone on the left side. Dr. Pean saw this patient for the first time in November, 1888; at that time the face was hideous to look upon; the left superior maxillary had the size of a new born child, the inferior maxillary was tumefied; the cheeks, eyelids and nose were pushed out; the buccal, orbital, nasal and pharyngo-nasal cavities were obstructed, the alveolar borders were thickened and the teeth were movable and displaced without allowing one to suppose that two of the teeth were missing by ectopia.

On Dec. 14, 1888, he performed the first operation; the anterior surface of the maxillary bone was exposed by Dr. Pean's method; the patient was placed in the dorsal position, with her body and neck elevated; sponges were kept in the back part of the mouth; the cheeks, nose and septum were pinched previous to operating; then a median section of the upper lip is made, as well as of the dorsum and root of the nose. The cheeks were then detached from their mouth attachment with the knife and scissors. As soon as the tumors were exposed their prominent surfaces were excised with a knife having a concave blade. The other portions of the tumor were removed with cutting forceps. During this part of the operation the superior maxillaries, the malar bones, the pterygoid processes, the naso-orbital septum and the floor of the orbit were all rapidly removed. At this moment it was found that the superior lobe of the tumor had engaged itself under the inferior compact part of the sphenoid bone, which was excised, and to the operator's great astonishment a little molar or bicuspid, placed transversely in the spongy portion of the bone, was found.

This led to the supposition that this dental heterotopia had been the cause ot the tumor, which was enucleated. Such an anomaly is almost unique in man; in animals, the horse in particular, such heteratopic odontoma are of somewhat frequent occurrence.

Six weeks later a second operation was performed to extirpate the inferior maxillary, by sectioning the soft parts from one angle to the other on a level with its inferior border, and dissection of the periosteum and of the tumor on both sides of the bone, together with bilateral section of the ascending rami and removal by pieces of the tumor which occupies the totality of the bone. The operation was completed by detaching from the symphysis the muscles which are there inserted, and cutting in the median line, the periosteum, which covers its inferior border. At this point a canine tooth was found placed in a transverse direction. This heterotopia is so much more interesting, as the teeth of the lower maxillary were complete in number.

The operation was a success; union by first intention took place, and after fourteen months there is no sign of the return of the trouble. Experience has demonstrated that tumors of this kind are extensively removed, as in the present instance, although there may be sarcomatous or myeloplaxic elements disseminated throughout the lamella and osseous fibrous tissue which compose it, they have very little tendency to recur.

Finally the author came to the following conclusions:

First. The total removal of the bony skeleton of the face can be successfully performed.

Second. Such an operation is indicated in cases of osteo-fibroma having for their origin dental heteropia, when these tumors occupy simultaneously both maxillaries. In such cases a permanent cure can be looked forward to.

Third. The deformation and functional troubles produced by it, can be easily corrected by prosthetic dentistry.

